# PARP-1 Val762Ala Polymorphism Is Associated with Risk of Cervical Carcinoma

**DOI:** 10.1371/journal.pone.0037446

**Published:** 2012-05-18

**Authors:** Feng Ye, Qi Cheng, Yuting Hu, Jing Zhang, Huaizeng Chen

**Affiliations:** 1 Women's Reproductive Health Key Laboratory of Zhejiang Province, Women's Hospital, School of Medicine, Zhejiang University, Hangzhou City, Zhejiang Province, China; 2 Department of Obstetrics and Gynecology, Women's Hospital, School of Medicine, Zhejiang University, Hangzhou City, Zhejiang Province, China,; Tsan Yuk Hospital, Hospital Authority, China

## Abstract

PARP-1 is a nuclear enzyme that plays an important role in DNA repair, recombination, proliferation and the genome stability. The PARP-1 Val762Ala polymorphism has been associated with increased risk of developing cancers of the prostate, esophagus and lung. The aim of this study was to determine whether the PARP-1 Val762Ala polymorphism is associated with the risk of cervical carcinoma. MA-PCR was used to genotype the PARP-1 Val762Ala polymorphism in 539 women with cervical carcinoma, 480 women with CIN and 800 controls. The genotyping method was confirmed by the DNA sequencing analysis. The PARP-1 Val762Ala polymorphism was not associated with the risk of CIN. However, women carrying the PARP-1 Ala762Ala genotype were significantly susceptible to cervical carcinoma (OR: 2.70, 95% CI: 1.47–3.70), and the similar results were also found in squamous cell carcinoma (OR: 2.56, 95% CI: 1.47–3.70). In HPV positive population, the PARP-1 Ala762Ala genotype was also associated with increased risk of cervical carcinoma (OR: 5.56, 95% CI: 2.08–14.3). Our results indicate that the PARP-1 Ala762Ala genotype increases the risk of cervical carcinoma.

## Introduction

Cervical carcinoma is one of the most common malignant diseases among women with nearly 500 000 women developing this disease each year [1], [2]. Cervical cancer is thought to develop through a multistep process involving virus, tumor suppressor genes, proto-oncogenes and immunological factors [3], [4]. It is well recognized that specific oncogenic human papillomaviruses(HPVs) are primary etiologic factor in cervical cancer [5], [6]. However, only a small fraction of HPV-infected lesions progress to cervical cancer or its precursor lesion cervical intraepithelial neoplasia(CIN), suggesting that other genetic factors may be involved in these processes [7]–[9].

DNA repair pathways exist in all cells for maintaining genome integrity [10], and mutations within these pathways can result in cancer [11]. Poly(ADP-ribose) polymerase 1(PARP-1) is a DNA strand break-sensing protein, and its activation is one of the early responses to DNA damage [12]. PARP-1 gene localizes to chromosome 1q41–42, consists of 23 exons and spans 47.3 kb [12]. It encodes a multifunctional nuclear protein, which consists of an N-terminal DNA binding domain, a central auto-modification domain and a C-terminal catalytic domain [13]. PARP-1 catalyzes poly(ADP-ribosyl)ation, an immediate DNA-damage dependent post-translational modification of itself, histones and other nuclear proteins, which is believed to play a multifunctional role in various cellular processes, including DNA-damage detection and repair, cell death pathways and mitotic apparatus function [14]. Research found that PARP-1^−/−^ mice show a higher susceptibility to carcinogenesis induced by alkylating agents(9). There are many identified single nucleotide polymorphism(SNP) in the PARP-1 gene, and some of which are reported to be implicated in carcinogenesis [15]–[18]. Of note, the SNP of PARP-1 Val762Ala (GTG/GCG) was associated with increased risk of various cancers [16], [17], [19].

To the best of our knowledge, there is still no research about the association between the SNP of PARP-1 Val762Ala(GTG/GCG) and cervical carcinoma. Therefore, in the current population-based case-control study, we investigated the genotype distributions of the PARP-1 Val762Ala(GTG/GCG) in patients with cervical carcinoma or CIN.

## Materials and Methods

### Study Population

All 1019 patients (including 539 cases cancer and 480 cases CIN) and 800 controls were unrelated ethnic Chinese women from Zhejiang Province. Of the 539 cases cancer was consisted of 489 squamous carcinoma, 47 adenocarcinoam, 1 undifferentiated carcinoma, 2 carcinosarcoma; FIGO stage Ia 53 cases, Ib 213 cases, IIa 133 cases, IIb 83cases, III 48 cases, IV 9 cases. Of the 480 cases CIN was consisted of 70 low grade cervical intraepithelial lesion(CIN 1), 410 high grade cervical intraepithelial lesion(CIN 2/3: 162 CIN 2, 248 CIN 3). The median ages of the cancer, CIN and control women were 45 (range, 25–81), 39 (range, 21–69) and 40.5 (range, 20–79) years, respectively. In the Hangzhou district, Patients with histologically confirmed primary cervical carcinoma or cervical dysplasia (CIN) at the Women's Hospital School of Medicine at Zhejiang University and the Cancer Hospital of Zhejiang Province were recruited; in the area outside of Hangzhou, a collaborative network of gynecological oncology teams in three areas (Lishui, Wenzhou and Shaoxing) of Zhejiang Province was established to identify new cases of cervical cancer or CIN at local hospitals between June 2004 and May 2006. The diagnosis date and tumor histology were recorded for all of the women, and there were no age, stage, histology, or prior therapy restrictions. During the same period, controls were randomly selected from healthy women seen for gynecologic examinations at the Women's Hospital School of Medicine at Zhejiang University. Control selection criteria included no positive cytological findings (TCT: Thinprep cytologic test), no history of cancer and immune disease, age matching to the patients and residence in Zhejiang Province during the gynecological examination. After providing signed informed consent, participants responded to a standard questionnaire about their sexual and reproductive history, including the number of sexual partners, the age at first intercourse, the age at first full-term pregnancy, parities (including full-term pregnancy and abortion at or after 28 weeks) and oral contraception used history. The study was approved by the local Medical Ethics Committees, and informed consent was obtained from all women included in the study(No. 2004002).

The blood samples of both patients and controls were collected. Of these, 689 patients (264 with cervical carcinoma and 425 with CIN) and 410 controls agreed to provide cervix brush-off samples for detection of HPV. The median ages of these HPV detecting cancer, CIN and control group were 42 (range, 25–76), 39 (range, 21–64) and 43 (range, 20–79) years, respectively.

### DNA extraction and Genotyping

Genomic DNA was extracted from the blood with phenol/chloroform and precipitated with cold ethanol. All genomic DNA samples were dissolved in water and stored at −20°C.

The SNP of PARP-1 Val762Ala(GTG/GCG) was determined by Modified polymerase chain reaction-mismatch amplification(MA-PCR). The two forward primers were 5′-TGCTCCTCCAGGCCAAGCT-3′and 5′-TGCTCCTCCAGGCCAAGCC-3′, which differ in only the last base, and the reverse primer was 5′-GAAACGCCCAAAGGTTCT-3′. The PCR product length was 330 bp.

The PCR was performed in a 25 ul reaction containing ∼50 ng of genomic DNA, 5.0 pmol of each primer, 0.2 mM of each deoxynucleoside triphosphate and 1.0 units of Taq DNA polymerase (Sangon, Shanghai, China). PCR was carried out under the following conditions: an initial denaturation at 94°C for 5 min, followed by 35 cycles of 94°C for 30 s, 57°C for 30 s, and 72°C for 45 s, and a final elongation step of 72°C for 10 min. The PCR products were examined by 1% agarose gel electrophoresis, stained with ethidium bromide and visualized with a Typhoon™ 9410 Imaging System (GE Healthcare, USA). To examine the reproducibility of the results, all samples were tested twice by two technicians and the results agreed for all masked duplicate sets. The results reproducibility of two technicians was one hundred percent.

To confirm the specificity of the MA-PCR for the polymorphisms, 20% of the samples were randomly selected (n = 200) and sequenced in a blinded manner. The forward and reverse primers for PCR sequencing were 5′-CCACCTGGGTGAGTCTGTCT-3′ and 5′-AGGCCTGACCCTGTTACCTT-3′, which produce a 212 bp fragment. Dye terminator DNA sequencing was performed in both directions using a BigDye Terminator Kit (Applied Biosystems, Foster City, CA, USA) at the ABI PRISM 3100 Genetic Analyzer (Applied Biosystems). PCR products and dye-terminated products were purified by ethanol/sodium acetate precipitation. Comparison of the two methods of analyzing polymorphisms did not identify any discordant pairs. These indicated that the MA-PCR specificity rate of detecting PARP-1 Val762Ala (GTG/GCG) was one hundred percent.

### HPV detection

HPV infection was identified using the Hybrid Capture II(HC II) assay (Digene Diagnostics Inc., Gaitherburg, MD, USA) using probe B, which contains a pool of full-length RNA probes specific for HPV 16, 18, 31, 33, 35, 39, 45, 51, 52, 56, 58, 59, and 68. Cervical sampling for HPV DNA was performed with the Digene Cervical Sampler, and the tests were classified according to the relative light units/positive control ratio, measured as the relative light units of the specimen divided by the mean relative light units of two positive controls.

### Statistical Analysis

Differences in frequencies of the genotypes and alleles between the cases of cervical carcinoma or CIN and controls were evaluated by the chi-square (χ^2^) test. Logistic regressions were applied to calculate the odds ratio(OR) and 95% confidence interval(CI), for the association between the genotypes and risk of cervical carcinoma. All statistical analyses were carried out using Statistical Package for Social Science software, version 11.5 (SPSS, Chicago, IL).

## Results

### Subject characteristics

The demographic and clinical characteristics of patients and controls are presented in Table 1. We analyzed 1019 patients (539 with cervical carcinoma and 480 with CIN) and 800 controls. There were no statistically significant differences between patients and controls in terms of number of sexual partners, age at the first intercourse, age at the first birth, smoking status and oral contraception used history. However, compared with controls, patients with CIN had a higher proportion of subjects less than 40 years of age (63.3% versus 50.0% in controls, P<0.001), while patients with cervical carcinoma had a lower proportion of subjects less than 40 years of age (40.1% versus 50.0% in controls, P<0.001). The proportion of subjects with the number of parities (>3) in both cervical carcinoma and CIN patients is more than that in controls (P<0.05). In addition, HPV infection rate was 31.0% in controls, 86.4% in patients with CIN, 88.3% in patients with cervical carcinoma, respectively, indicating that higher HPV infection rate in patients (P<0.001).

**Table 1:** Frequency distribution of selected characteristics by case control status.

Variable		Control(%),total = 800	CIN(%),total = 480	χ^2^ *	*P*	Carcinoma(%),total = 539	χ^2^ *	P
Age	≤40	400 (50.0)	304(63.3)	**21.549**	**<0.001**	216(40.1)	**12.772**	**<0.001**
	>40	400(50.0)	176(36.7)			323(59.9)		
Number of sexual partners	≤1	642(80.3)	381(79.4)	0.143	0.719	422(78.3)	0.756	0.408
	>1	158(19.7)	99(20.6)			117(21.7)		
Age at the first intercourse	≤20 years	236(29.5)	153(31.9)	0.800	0.380	174(32.3)	1.173	0.304
	>20 years	564(70.5)	327(68.1)			365(67.7)		
Number of parities	≤3	361(45.1)	186(36.7)	**4.909**	**0.030**	181(33.6)	**17.813**	**<0.001**
	>3	439(54.9)	294(63.3)			358(66.4)		
Age at the first birth	≤22years	155(19.4)	105(21.9)	1.051	0.315	120(22.3)	1.646	0.214
	>22 years	645(80.6)	375(78.1)			419(77.7)		
Smoking status	smoker	2(0.3)	2(0.4)	0.268	0.633	4(0.7)	1.748	0.224
	nonsmoker	798(99.7)	478(99.6)			535(99.3)		
HR-HPV infection	Positive	127(31.0)	367(86.4)	**264.876**	**<0.001**	233(88.3)	**211.764**	**<0.001**
	Negative	283(69.0)	58(13.6)			31(11.7)		
oral contraceptive	Yes	14(1.8)	10(2.1)	0.181	0.675	12(2.2)	0.384	0.550
	No	786(98.3)	470(97.9)			527(97.8)		

* Two-sided χ^2^ test.

### Polymorphism of PARP-1 and the risk of CIN

All genotypic distributions were consistent with that expected in the Hardy–Weinberg model (χ^2^ = 4.571, *P* = 0.102). Table 2 showed that the frequency of the PARP-1 Ala762Ala(GCG/GCG), Val762Ala(GTG/GCG) and Val762Val(GTG/GTG) genotypes were 8.6%, 60.0% and 31.4% in patients with CIN 1, and 8.5%,60.7%,30.7% in patients with CIN 2/3, which was not different with that in controls (8.5%, 59.4%, 32.1%). Also, in the HPV positive subgroup, there was no difference in the genotypic distribution of PARP-1 Val762Ala(GTG/GCG) between controls and patients with CIN (Table 3). Likewise, patients with CIN had the same frequency of the PARP-1 Ala762Ala(GCG/GCG), Val762Ala(GTG/GCG) and Val762Val(GTG/GTG) genotypes as controls in different sexual or reproductive histories subgroup (Table 4). Thus, the Ala762Ala(GCG/GCG) genotype of PARP-1 was not a significantly increased risk of developing CIN.

**Table 2:** Analysis of association between the polymorphisms and risk of CIN.

Genotypes*	Control(%),total = 800	CIN 1(%),total = 70	adjusted OR**(95% CI)	*P*	CIN 2/3(%),total = 410	adjusted OR**(95% CI)	*P*
TT	257(32.1)	22(31.4)	1		126(30.7)	1	
TC	475(59.4)	42(60.0)	1.03(0.60–1.77)	0.906	249(60.7)	1.07(0.82–1.39)	0.617
CC	68(8.5)	6(8.6)	1.03(0.40–2.64)	0.950	35(8.5)	1.05(0.66–1.66)	0.836
TC or CC	543(67.9)	48(68.6)	1.03(0.61–1.75)	0.905	284(69.2)	1.07(0.83–1.38)	0.622

* CC: PARP-1 Ala762Ala(GCG/GCG); TC: Val762Ala(GTG/GCG); TT: Val762Val(GTG/GTG).

** all P-values adjusted for age.

**Table 3:** According to HPV infection status(HPV positive) stratified analysis of the association between genotypes and risk of cervical carcinoma and CIN.

Genotypes*	Control(%),total = 127	CIN(%),total = 367	adjusted OR**(95% CI)	*P*	Carcinomas(%),total = 233	adjusted OR**(95% CI)	*P*
TT	46(36.2)	115(31.3)	1.00		41(17.6)	1.00	
TC	76(59.8)	225(61.3)	1.18(0.77–1.82)	0.440	125(53.6)	**1.85(1.11–3.07)**	**0.018**
CC	5(3.9)	27(7.4)	2.17(0.78–5.88)	0.137	67(28.8)	**5.56(2.08–14.30)**	**0.001**
TC or CC	81(63.8)	252(68.7)	1.24(0.82–1.90)	0.312	192(82.4)	**2.66(1.62–4.36)**	**<0.001**

* CC: PARP-1 Ala762Ala(GCG/GCG); TC: Val762Ala(GTG/GCG); TT: Val762Val(GTG/GTG).

** all P-values adjusted for age.

**Table 4:** According to different sexual or reproductive histories subgroup stratified analyses between the genotypes and CIN risk.

High risk exposure	Controls(%)		CIN cases(%)		Adjusted OR**(95% CI)	P
	TT*	TC or CC*	TT*	TC or CC*		
Number of sexual partners						
≤1	204(31.8)	438(68.2)	123(32.3)	258(67.7)	0.98(0.75–1.28)	0.866
>1	53(33.5)	105(66.5)	25(25.3)	74(74.7)	1.49(0.85–2.62)	0.161
Age at the first intercourse						
≤20 years	73(30.9)	163(69.1)	50(32.7)	103(67.3)	0.92(0.60–1.43)	0.717
>20 years	184(32.6)	380(67.4)	98(30.0)	229(70.0)	1.13(0.84–1.52)	0.412
Number of parities						
≤3	118(32.7)	243(67.3)	63(33.9)	123(66.1)	0.95(0.65–1.38)	0.781
>3	139(31.7)	300(68.3)	84(28.7)	209(71.3)	1.15(0.83–1.59)	0.389
Age at the first parity						
≤22 years	45(29.0)	110(71.0)	35(33.7)	69(66.3)	0.81(0.47–1.38)	0.430
>22 years	212(32.9)	433(67.1)	112(29.9)	262(70.1)	1.15(0.87–1.51)	0.335
Oral contraceptive						
Yes	3(21.4)	11(78.6)	2(20.0)	8(80.0)	1.09(0.15–8.12)	0.932
No	254(32.3)	532(67.7)	146(31.1)	324(68.9)	1.06(0.83–1.36)	0.645

* CC: PARP-1 Ala762Ala(GCG/GCG); TC: Val762Ala(GTG/GCG); TT: Val762Val(GTG/GTG).

** all P-values adjusted for age.

### Polymorphism of PARP-1 and the risk of cervical carcinoma

As shown in Table 5, the frequency of the PARP-1 Ala762Ala(GCG/GCG),Val762Ala(GTG/GCG) and Val762Val(GTG/GTG) genotypes were 8.5%, 59.4% and 32.1%, respectively in controls, 19.3%, 52.5% and 28.2% in patients with cervical carcinoma. PARP-1 Ala762Ala(GCG/GCG) carriers in patients with cervical carcinoma were significantly higher than those in controls (OR: 2.70, 95% CI: 1.47–3.70; P<0.001). The distributions of these genotype frequencies in subjects with squamous cell carcinoma were the same as those in total carcinoma patients (OR: 2.56, 95% CI: 1.47–3.70; P<0.001). Table 3 showed that in the HPV positive subgroup, subjects with the PARP-1 Ala762Ala(GCG/GCG) genotype in patients with cervical carcinoma are significantly more than those in controls (OR: 5.56, 95% CI: 2.08–14.3; P<0.01); P<0.01]. What is more, patients with cervical carcinoma had a higher proportion of subjects with the PARP-1 Ala762Ala(GCG/GCG) or Val762Ala(GTG/GCG) genotype compared to controls in terms of the number of sex partners (Table 6). These results indicate that women carrying the PARP-1 Ala762Ala(GCG/GCG) genotype had an increased risk for cervical carcinoma compared with those carrying the PARP-1 Val762Val(GTG/GTG) genotype.

**Table 5:** Analysis of association between the polymorphisms and risk of cervical carcinoma.

Genotypes*	Control(%),total = 800	Carcinomas(%),total = 539	adjusted OR**(95% CI)	*P*	Squamous cell carcinoma(%),total = 489	adjusted OR**(95% CI)	*P*
TT	257(32.1)	152(28.2)	1.00		139(28.4)	1.00	
TC	475(59.4)	283(52.5)	0.39(0.79–1.29)	0.594	255(52.1)	0.84(0.67–1.05)	0.135
CC	68(8.5)	104(19.3)	**2.70(1.47–3.70)**	**<0.001**	95 (19.5)	**2.56(1.47–3.70)**	**<0.001**
TC or CC	543(67.9)	387(71.8)	1.20(0.95–1.53)	0.127	350(71.6)	1.19(0.93–0.52)	0.163

* CC: PARP-1 Ala762Ala(GCG/GCG); TC: Val762Ala(GTG/GCG); TT: Val762Val(GTG/GTG).

** all P-values adjusted for age.

**Table 6:** According to different sexual or reproductive histories subgroup stratified analyses between the genotypes and cervical carcinoma risk.

High risk exposure	Controls(%)		Carcinoma cases(%)		Adjusted OR**(95% CI)	P
	TT*	TC or CC*	TT*	TC or CC*		
Number of sexual partners						
≤1	204(31.8)	438(68.2)	121(28.7)	301(71.3)	**1.46(1.11–1.92)**	**0.007**
>1	53(33.5)	105(66.5)	31(26.5)	86(73.5)	1.40(0.83–2.37)	0.210
Age at the first intercourse						
≤20 years	73(30.9)	163(69.1)	39(22.4)	135(77.6)	1.55(0.99–2.43)	0.057
>20 years	184(32.6)	380(67.4)	113(31.0)	252(68.9)	1.08(0.81–1.43)	0.595
Number of parities						
≤3	118(32.7)	243(67.3)	53(29.3)	128(70.7)	1.17(0.80–1.73)	0.421
>3	139(31.7)	300(68.3)	99(27.7)	259(72.3)	1.21(0.89–1.65)	0.219
Age at the first parity						
≤22 years	45(29.0)	110(71.0)	35(29.2)	85(70.8)	0.99(0.59–1.68)	0.981
>22 years	212(32.9)	433(67.1)	117(27.9)	302(72.1)	1.26(0.97–1.65)	0.089
Oral contraceptive						
Yes	3(21.4)	11(78.6)	3(25.0)	9(75.0)	0.82(0.13–5.08)	0.830
No	254(32.3)	532(67.7)	149(28.3)	378(71.7)	1.21(0.95–1.54)	0.120

* CC: PARP-1 Ala762Ala(GCG/GCG); TC: Val762Ala(GTG/GCG); TT: Val762Val(GTG/GTG).

** all P-values adjusted for age.

## Discussion

DNA repair systems play a key role in protecting against carcinogenesis and genetic defect in DNA repair can cause human cancer [11]. Through repairing DNA damage and maintaining genetic stability, PARP-1 has played an important role in prevention of carcinogenesis. PARP-1 deficiency has enhanced tumorigenesis and widened the tumor spectrum in tumor protein p53-deficient mice [20]. Treatment with the alkylating agent, azoxymethane, enhanced the frequency of tumor development in the colon and liver of PARP-1^−/−^ mice [21]. The PARP-1 Val762Ala(GTG/GCG) polymorphism has been implicated in cancer susceptibility. It is associated with increased risk of prostate cancer, esophageal squamous cell carcinoma, smoking-related lung cancer and gastric cardia cancer [16]–[17], [19]–[20]. In this study, we found for the first time that the PARP-1 Ala762Ala(GCG/GCG) genotype significantly contributes to cervical carcinoma susceptibility, which further extend the important role of PARP-1 in carcinogenesis. Surprisingly, the PARP-1 Ala762Ala(GCG/GCG) genotype is not associated with increased risk of developing CIN. The majority of subjects with CIN were less than 40 years of age, while those with cervical carcinoma were more than 40 years of age. Thus, PARP-1 dysfunction maybe induces carcinogenesis after the development of CIN.

Many previous studies have showed that HPV positive subjects have a significantly increased risk of developing cervical carcinoma compared with HPV negative ones [22]–[24]. Our results support that HPV infection is a definite risk factor for cervical carcinoma, because patients with cervical carcinoma had an about 2-fold increased HPV infection rate compared with controls. In addition, HPV positive subjects with the PARP-1 Ala762Ala(GCG/GCG) genotype had stronger association with risk of cervical carcinoma, implying that interaction of HPV infection with the genetic variation of host jointly contributes to the cervical carcinogenesis.

The increased risk of cervical carcinoma in subjects with the PARP-1 Ala762Ala(GCG/GCG) genotype is likely attributable to the reduction of PARP-1 activity. PARP-1 catalyze extensive polymerization of ADP-ribose from its substrate niconamide adenine dinucleotide(NAD^+^) to nuclear proteins, including histones, X-ray repair cross-complementing factor-1(XRCC-1), nuclear factor kappa-light-chain-enhancer of activated B cells (NF-κB), p53 and PARP-1 itself [14], [25]. Residue 762 might be implicated in the binding of substrate NAD^+^, and crystal structure of the catalytic domain of human PARP-1 reveals that 762Val is located in the fifth helix of the PARP-1 N-terminal regulatory subdomain, facing the pocket of the active site [26]. The recent research show that PARP-1 762Ala displayed 57.2% of the activity of PARP-1 762Val for auto-poly(ADP-ribosyl)ation and 61.9% of the activity of PARP-1 762Val for trans-poly(ADP-ribosyl)ation of histone H1, indicating that the PARP-1 Ala762 reduces the enzymatic activity of PARP-1. Furthermore, Lockett et al. report that the PARP-1 activity with 762Ala is decreased compared to the PARP-1 762Val, and the PARP-1 Ala762Ala(GCG/GCG) genotype is associated with an increased risk for prostate cancer in Caucasian subjects [16]. Thus, 762Val-to-Ala substitution associated with reduced poly(ADP-ribosyl)ation activity, decreases cellular repair function and therefore causes genome instability, leading to cervical carcinoma susceptibility.

In summary, our study firstly shows a significant association between the PARP-1 Ala762Ala(GCG/GCG) genotype and increased risk of cervical carcinoma. Our findings suggest that PARP-1 dysfunction may play an important role in the development of cervical carcinoma.
